# Flower color as a model system for studies of plant evo-devo

**DOI:** 10.3389/fpls.2013.00321

**Published:** 2013-08-20

**Authors:** James M. Sobel, Matthew A. Streisfeld

**Affiliations:** Institute of Ecology and Evolution, University of OregonEugene, OR, USA

**Keywords:** anthocyanin, flower color, R2R3-MYB, predictability, co-option, pleiotropy

## Abstract

Even though pigmentation traits have had substantial impacts on the field of animal evolutionary developmental biology, they have played only relatively minor roles in plant evo-devo. This is surprising given the often direct connection between flower color and fitness variation mediated through the effects of pollinators. At the same time, ecological and evolutionary genetic studies have utilized the molecular resources available for the anthocyanin pathway to generate several examples of the molecular basis of putatively adaptive transitions in flower color. Despite this opportunity to synthesize experimental approaches in ecology, evolution, and developmental biology, the investigation of many fundamental questions in evo-devo using this powerful model is only at its earliest stages. For example, a long-standing question is whether predictable genetic changes accompany the repeated evolution of a trait. Due to the conserved nature of the biochemical and regulatory control of anthocyanin biosynthesis, it has become possible to determine whether, and under what circumstances, different types of mutations responsible for flower color variation are preferentially targeted by natural selection. In addition, because plants use anthocyanin and related compounds in vegetative tissue for other important physiological functions, the identification of naturally occurring transitions from unpigmented to pigmented flowers provides the opportunity to examine the mechanisms by which regulatory networks are co-opted into new developmental domains. Here, we review what is known about the ecological and molecular basis of anthocyanic flower color transitions in natural systems, focusing on the evolutionary and developmental features involved. In doing so, we provide suggestions for future work on this trait and suggest that there is still much to be learned from the evolutionary development of flower color transitions in nature.

## Introduction

Adaptation is the driving force behind the amazing diversity of life on this planet, and the past several years have seen an explosion of experimental studies seeking to identify the genetic and molecular bases of adaptive traits (Hoekstra et al., 2006; Rebeiz et al., 2009; Wittkopp et al., 2009; Chan et al., 2010). An important question that emerges from these studies is whether the genetic changes contributing to phenotypic evolution are predictable. In other words, does natural selection preferentially target specific genes—or certain types of mutations—during adaptation? Such predictability suggests that phenotypic change occurs via non-random sampling of potential genetic variation. This may indicate substantial constraint on the process of adaptation, as the epistatic and pleiotropic structure of developmental regulatory networks limits the mutations that result in evolutionary transitions (Stern, 2011). As a consequence, the question of predictability has been of particular interest in the evo-devo literature where multiple surveys have been conducted across broad suites of traits in plants and animals in an attempt to determine whether certain types of evolutionarily relevant mutations are more common than others (Stern, 2000; Carroll, 2005; Hoekstra and Coyne, 2007; Prud'homme et al., 2007; Wray, 2007; Carroll, 2008; Lynch and Wagner, 2008; Stern and Orgogozo, 2008; Wagner and Lynch, 2008; Stern and Orgogozo, 2009).

The independent evolution of the same trait among populations or species is known as phenotypic convergence. Convergence has been shown to occur at different phylogenetic distances. For example, closely related populations within the species *Arabidopsis thaliana* have independently evolved early-flowering phenotypes (Johanson et al., 2000), while across much broader time scales, at least nine different plant families have independently evolved succulence in arid environments (e.g., Cactaceae and Euphorbiaceae) (Mauseth, 2004). In many of these examples, compelling evidence for adaptation exists because convergence accompanies similar environmental pressures, suggesting that natural selection independently drove the evolution of similar phenotypes. Moreover, from a mechanistic perspective, multiple origins of the same trait offer naturally occurring replicates of the evolutionary process and provide us with an opportunity to explore the types of constraints that dictate the course of phenotypic evolution (Kopp, 2009).

When the genetic basis for this phenomenon is characterized, it has been found in a variety of systems that changes in the same gene, sometimes involving the same substitution, are responsible for the independent origins of the same character. However, in other systems, independent phenotypic transitions have distinct genetic and molecular mechanisms (Arendt and Reznick, 2008; Martin and Orgogozo, 2013). The presence of genetic convergence suggests that evolution can at times be predictable under certain environmental, genetic, and phylogenetic contexts. This can occur due to constraints in the available variation upon which natural selection can act, which can lead to predictable evolutionary outcomes. Therefore, there is substantial interest in the factors that determine how and why convergence at the genetic level accompanies convergence at the phenotypic level (Manceau et al., 2010).

Plant evo-devo has focused primarily on the evolution of the regulatory networks involved in transitions in floral organ identity (Kramer and Irish, 1999; Becker and Theissen, 2003), floral symmetry (Preston and Hileman, 2009), and flowering time (Caicedo et al., 2004). Plant pigmentation (especially of flowers) frequently has been investigated from an evolutionary genetics perspective (Rausher, 2008), with several excellent examples where the genetic and molecular basis of evolutionary transitions in flower color has been characterized. However, this trait has yet to be widely adopted by plant evolutionary developmental biologists. This is perhaps surprising given the prominence of pigmentation traits in the study of animal evo-devo (e.g., Gompel et al., 2005), as many of the big questions in evo-devo are approachable by examining general patterns among these case studies. These include the roles of developmental constraint, redundancy in the genome, the origin of novelty, and the relationship between micro- and macroevolution (Jenner and Wills, 2007). Transitions in floral color are common among the angiosperms, with sister species frequently showing variation in pigment intensity and hue (Rausher, 2008). The anthocyanin pigments are the most common floral pigments and are produced via highly conserved structural and regulatory components (Grotewold, 2006). Differences in flower color among populations or closely related species are often suspected to be directly related to plant fitness, as mediated through pollinator preferences (Bradshaw and Schemske, 2003; Streisfeld et al., 2013). Therefore, the phylogenetically conserved anthocyanin pathway and its associated adaptive phenotypes represent excellent opportunities to merge evo-devo and ecological/evolutionary genetics to investigate the predictability of genetic changes associated with flower color transitions.

Flower color transitions can involve gains or losses of pigment, but how the directionality of flower color change impacts predictability is largely unexplored. Therefore, our primary goal in this review is to explore how this phylogenetic context affects the predictability of flower color change. While flower color transitions may occur via random genetic drift or indirect selection, we focus the review on instances where flower color is presumed to be a direct target of selection via pollinator visitation, physiological traits, or pleiotropic consequences. We also examine the role that population frequency plays on predictability, as flower color changes can occur in nature both as within population polymorphisms and as fixed differences between populations and species. Based on our knowledge of the biochemistry and regulation of anthocyanins, we present experimental predictions for the types of mutations expected to underlie different evolutionary transitions in flower color. We then review the fit of experimental genetic data to our predictions and examine possible mechanisms that may account for the current patterns. For situations where no empirical genetic data exist, we provide suggestions for future work that will make formal tests of these hypotheses possible. We stress that many of the outstanding questions in this field can only be assessed by characterizing multiple examples of the genetic basis of this ecologically important trait.

## Predictability and phylogenetic context

As introduced above, we define genetic change to be predictable when mutations of one type occur more frequently than others to confer the same phenotypic transition. The term “substitution bias” has been used to reflect this type of predictability for fixed differences between populations (Streisfeld and Rausher, 2011). In particular, when the same phenotype fixes repeatedly across lineages, a substitution bias exists if one type of genetic change is responsible for this convergence more frequently than other types. It is generally accepted that there are two processes that can generate substitution bias: a particular type of mutation has a higher mutation rate (“mutation bias”) or a higher probability of fixation once the mutation arises (“fixation bias”).

To understand how differences in mutation and/or fixation biases influence substitution bias, imagine a situation where multiple, independent lineages have evolved the derived phenotype “*B*,” and mutations in multiple categories (*1, 2, 3,…., n*) can generate this phenotype (see Streisfeld and Rausher, 2011 for formal description). A mutation bias exists if mutations in one or more categories occur more frequently than others, as can occur via differences in mutational target size (e.g., Ossowski et al., 2010). By contrast, a fixation bias occurs when there are different probabilities of fixation among these categories. Fixation probabilities are proportional to the net selection coefficient (*s*), which consists of the difference between the strength of selection favoring the advantageous phenotype (*s*_*a*_) and any deleterious pleiotropic consequences of the same mutation (*s*_*p*_; in other words: *s* = *s*_*a*_ − *s*_*p*_). Because selection operates on phenotypes and not genotypes, *s*_*a*_ will be similar for all mutations causing phenotype *B* in a given environment. However, some mutations will have fewer negative pleiotropic consequences and thus a higher net selection coefficient. Those mutations with the highest *s* are thus the most likely to be fixed. This implies that the predictability of genetic evolution is closely tied to the extent of deleterious pleiotropy associated with the mutations capable of generating phenotype “*B*.”

While this framework exists for understanding and assessing whether genetic change is predictable, direct calculation of mutation and fixation biases are exceedingly difficult, primarily because they require great effort to monitor rates of spontaneous mutations and the subsequent fixation rate of those mutations in nature. Therefore, previous attempts at examining predictability have focused mostly on determining whether substitution biases exist for different types of mutations (e.g., coding vs. *cis-regulatory* mutations) across a broad spectrum of traits from both plants and animals (Carroll, 2005; Hoekstra and Coyne, 2007; Wray, 2007; Stern and Orgogozo, 2008). However, substitution bias is most effectively estimated by focusing on the repeated evolution of a single phenotypic trait, because each trait has a unique mutational spectrum (Kopp, 2009; Streisfeld and Rausher, 2011; Conte et al., 2012; Martin and Orgogozo, 2013). Indeed, the repeated evolution of similar flower color phenotypes has allowed for a rigorous analysis of not only substitution biases, but also the mutation and fixation biases that contribute to substitution bias (Streisfeld and Rausher, 2011).

Two underappreciated factors that can influence the likelihood of predictability are the directionality of the phenotypic transition and the population frequency in which polymorphisms exist. Because losses of floral anthocyanins are believed to occur more frequently than gains, previous work has focused exclusively on losses (Streisfeld and Rausher, 2011). However, several examples now document gains in floral pigmentation between populations, which may impact the spectrum of mutations capable of generating the phenotype. Similarly, the literature on predictability is based largely on differences that occur between species. However, it has been argued that the types of mutations involved in phenotypic change can vary due to the efficacy of natural selection across different evolutionary timescales (Stern and Orgogozo, 2008). Ultimately, those mutations that are universally advantageous in a given environment have the greatest potential to become fixed and persist between populations or species. However, examples of convergence in floral pigmentation are common both as within population polymorphisms as well as fixed differences between populations. Nevertheless, little attention has been paid to how the types of mutations that may be responsible for these convergent phenotypes will vary depending on the frequency of the derived flower color. Thus, the primary goal of this review is to examine how directionality and population frequency influence the predictability of the genetic changes associated with evolutionary transitions in flower color.

## Anthocyanin biosynthesis and flower color

We begin with a brief introduction of the genetics, biochemistry, and regulation of the anthocyanin pigmentation pathway. Our goal here is to provide context for the sections that follow as opposed to presenting an exhaustive review of the field. For more details on these topics, we refer the reader to several excellent, recent reviews on the subject (Holton and Cornish, 1995; Grotewold, 2006; Feller et al., 2011; Hichri et al., 2011; Davies et al., 2012).

Anthocyanins are common floral pigments that give rise to blue, purple, and red colors, and the flavonoid biosynthetic pathway responsible for anthocyanin pigmentation is highly conserved (Holton and Cornish, 1995; Winkel-Shirley, 2002). Flower color appears to be an evolutionarily labile trait, and transitions in floral anthocyanin pigmentation typically are of one of two types: (1) changes in pigment intensity, which are generally determined by the concentration of pigment in floral tissue, and (2) changes in floral hue, which are often determined by the types of pigments and co-pigments present in a flower. In some circumstances, shifts in both intensity and hue have been reported (Hopkins and Rausher, 2011). Because the predictability of floral hue transitions have been the focus of previous work (see Streisfeld and Rausher, 2011; Wessinger and Rausher, 2012), we focus here on transitions in pigment intensity.

**Figure 1:**
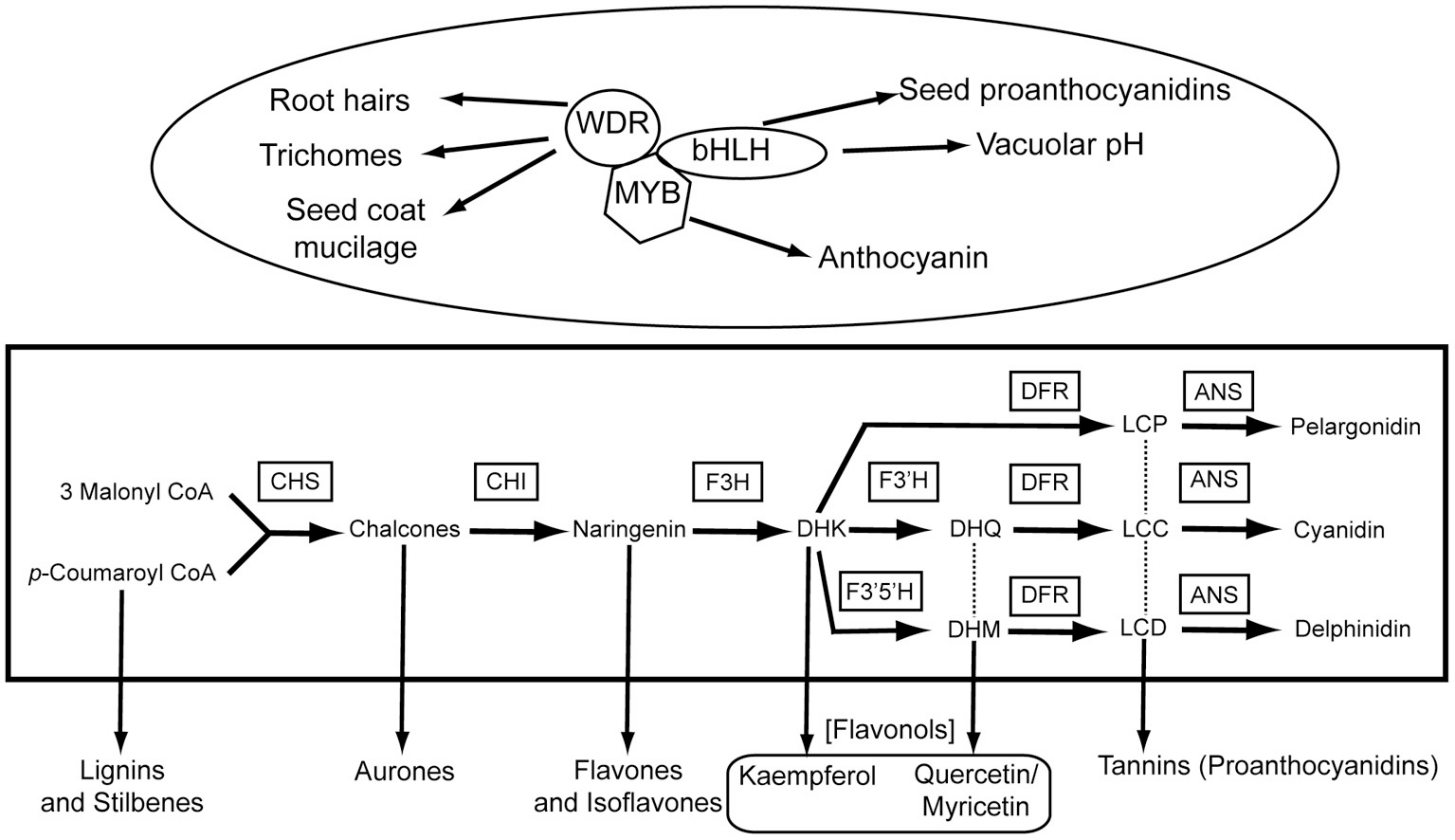
**A schematic of the core anthocyanin biosynthetic pathway (ABP; rectangle) and its regulation (ellipse).** The core enzymes of the ABP are listed as abbreviations above the arrows in boxes, while the products of each enzymatic reaction are listed next to the arrows. The hexagon labeled “MYB” can refer to either R2R3-MYB or R3-MYB proteins. The MBW complex is known to regulate the ABP, as well as several other aspects of epidermal cell differentiation (connected by arrows). The precise ABP targets of the MBW complex vary among species, so no connections are drawn. For simplicity, various ABP repressors are not shown. Additional non-anthocyaninic flavonoids produced as side branches of the core ABP are connected by arrows to the ABP. Abbreviations: CHS, chalcone synthase; CHI, chalcone isomerase; F3H, flavonoid 3-hydroxylase; F3′H, flavonoid 3′ hydroxylase; F3′5′H, flavonoid 3′ 5′ hydroxylase; DFR, dihydroflavonol 4-reductase; ANS, anthocyanidin synthase.

With a few exceptions, all anthocyanin pigments are derived from one of three anthocyanin precursors: pelargonidin, cyanidin, and delphinidin (Grotewold, 2006). These precursors are produced by alternative branches of the pathway (Figure 1). At least six different enzymatic reactions must occur to produce pigments, and the genes coding for these enzymes have been characterized in several species (Figure 1; Holton and Cornish, 1995). These enzymes also serve as branching points for the non-pigmented flavonoids, including lignins, flavonols, and other phenolic compounds such as tannins that play important physiological roles in plant stress responses (Winkel-Shirley, 2002).

In addition to the structural backbone of the pathway, much is known about the regulation of these enzymes. In all species that have been examined, coordinated gene regulation of multiple pathway enzymes occurs at the level of transcription via the interaction of *cis*-regulatory elements in pathway genes and their transcription factor complexes (Quattrocchio et al., 2006). Detailed investigation from Arabidopsis, maize, and Petunia has provided a nearly complete description of an interacting transcription factor complex that is made up of proteins encoded by members from three large gene families: the R2R3-MYB, basic helix-loop-helix (bHLH) and WD40-repeat (WDR) families (Broun, 2005; Koes et al., 2005; Ramsay and Glover, 2005). Hereafter, we refer to this complex as the MBW complex, which has become a paradigm for our understanding of coordinated gene regulation in plants (Feller et al., 2011). Moreover, MYB-domain proteins lacking one of the canonical repeat domains associated with DNA binding (known as R3-MYBs), have been shown to repress anthocyanins and other flavonoids in vegetative tissues (Quattrocchio et al., 2006; Matsui et al., 2008; Dubos et al., 2010; Albert et al., 2011). However, because the full range of factors that can repress the pathway is not fully characterized, we focus primarily on the role of the MBW complex in regulating differences in anthocyanin pigment intensity.

In addition to its conserved role in regulating the expression of anthocyanin pigmentation, the MBW complex also controls alternative developmental pathways that affect various aspects of epidermal cell fate, including the initiation of trichomes, alteration of vacuolar pH, production of root hairs, seed coat mucilage, and proanthocyanidin synthesis in seeds (Broun, 2005; Koes et al., 2005; Ramsay and Glover, 2005). As has been demonstrated in Arabidopsis, a single WDR protein (TTG-1) and a limited number of bHLH proteins (e.g., GL3, EGL3, TT8) are responsible for regulating all of these functions (Broun, 2005). The specificity of each function appears to be the result of the particular R2R3-MYB protein that joins the complex (e.g., TT2, PAP1, PAP2, WER, GL1; Ramsay and Glover, 2005; Schwinn et al., 2006; Dubos et al., 2010).

R2R3-MYBs represent one of the largest families of plant transcriptional regulators, and they appear to have undergone a rapid expansion during the evolution of land plants (Feller et al., 2011). For example, different copies of R2R3-MYB proteins are known to confer tissue- and cell-specific anthocyanin regulation in several species, whereas other copies are responsible for trichome initiation and proanthocyanidin synthesis in seeds (Broun, 2005; Koes et al., 2005; Morita et al., 2006; Quattrocchio et al., 2006; Schwinn et al., 2006; Gonzalez et al., 2008). This pattern suggests that duplication of R2R3-MYB proteins has allowed different copies to become specialized in their expression and developmental control of specific traits. By contrast, this specialization does not appear to have occurred for WDR or bHLH proteins (Koes et al., 2005; Ramsay and Glover, 2005; Morita et al., 2006), which has implications for the predictability of the genetic changes responsible for flower color evolution (see below).

Based on this understanding of the structural and regulatory components of this pathway, we can specify the set of possible mutation types that can give rise to repeated transitions in floral pigment intensity. Specifically, to obtain a change in pigment intensity, the amount of flux through the pathway must be altered. In principle, such alteration may be caused in any of four ways: (i) by *cis*-regulatory changes to enzyme-coding genes that directly affect enzymatic expression; (ii) by coding-sequence mutations in enzyme-coding genes; (iii) by *cis*-regulatory changes to anthocyanin transcription factors; or (iv) by coding-sequence mutations to anthocyanin transcription factors (Streisfeld and Rausher, 2011). Previous investigation has shown that functional deactivation or reduced expression of any of the pathway enzymes or transcription factors can result in a loss of pigmentation (Grotewold, 2006), though as we discuss below, this set of mutations is reduced if the transition involves a gain of floral pigmentation.

## Evolutionary gains and losses

It is important to recognize that loss vs. gain of function can occur at either the phenotypic or genetic level, and no correspondence between the two is necessary. For example, loss of pigment production can be achieved either through functional inactivation of enzymes or transcription factors (loss of function at the genetic level) or through recruitment of an inhibitor of anthocyanin production (gain of function). We focus this review at the phenotypic level, with gains and losses referring to the intensity of floral anthocyanin pigmentation. The direction of these transitions is typically determined based on phylogenetic comparative methods. Sophisticated tools that jointly estimate the transition and diversification rates have been developed recently for this purpose (Maddison et al., 2007; Fitzjohn et al., 2009). However, for those groups where characterization of the genetic basis for evolutionary transitions in floral pigment intensity exists, these methods have unfortunately not yet been employed. Therefore, acknowledging the potential limitations, we define “losses” and “gains” in this review largely in a parsimony framework, where we consider a “***gain***” when an anthocyanic flower occurs in a clade dominated by unpigmented flowers, and a “***loss***” when flowers completely lack or have significant reductions in floral anthocyanins within a clade of mostly pigmented flowers.

## Evolutionary losses of floral pigment intensity

### Experimental predictions

We begin by describing our predictions for the types of mutations that should predominate among evolutionary losses of floral pigment intensity. To do so, we distinguish between losses that are fixed between populations or species and those that segregate as polymorphisms within populations. In their survey of the molecular changes contributing to phenotypic evolution in plants and animals, Stern and Orgogozo (2008, 2009) observed that *cis*-regulatory changes dominated transitions in morphology between species, but phenotypic variation within species was caused by an elevated frequency of null coding mutations. To explain this observation, they argued that alleles that persist between species likely have been exposed to heterogeneous environmental and selective pressures over a longer time-scale than alleles that vary within species. Because the probability of fixation of a mutation is proportional to its net selective advantage, only those mutations with the fewest negative pleiotropic effects should be favored over the long term (Orr, 2000; Otto, 2004). These results suggest that substantial selective filters generate non-random biases in the spectrum of mutations found between species compared to those that arise and segregate within species (Gompel and Prud'homme, 2009). However, the definition of species can vary tremendously between different taxonomic treatments in many plant groups, and the delineation of species boundaries is often an arbitrary choice of which taxonomy is followed. Therefore, flower color polymorphisms that would be defined as within-species polymorphisms following one taxonomy would be considered interspecific in another. However, we expect that flower color variants that have fixed between populations will be exposed to similar selective filters regardless of whether those differentiated populations are considered different species or not. Therefore, we separate examples and hypotheses based on the categories 1) segregating (non-fixed) polymorphisms, and 2) fixed differences between populations or species.

As a consequence of the extensive investigation into the function and regulation of the genes comprising the anthocyanin pathway (Grotewold, 2006), we have a reasonable ability to make predictions about the genetic targets responsible for losses in flower pigment intensity. These expectations derive from our knowledge of the potential deleterious pleiotropic effects of these mutations on fitness. For example, as discussed above, the pathway enzymes responsible for anthocyanin biosynthesis are also required to synthesize other flavonoids. Likewise, the bHLH and WDR proteins that regulate the pathway enzymes have broad expression domains and are responsible for regulating multiple developmental processes in different tissues. Thus, while coding mutations in the pathway enzymes and these transcription factors are expected to arise and segregate in populations, extensive deleterious pleiotropic effects of these mutations would likely prevent their fixation.

By contrast, *cis*-regulatory mutations in any of the pathway genes and/or transcription factors are capable of specifically altering floral pigment intensity without impacting flavonoid biosynthesis elsewhere in the plant. Therefore, *cis*-regulatory mutations are believed to harbor fewer deleterious pleiotropic effects. Similarly, individual copies of R2R3-MYBs are often tissue-specific, such that a single copy may regulate pigment intensity specifically in flowers while another copy regulates pigmentation in the vegetative tissues. Therefore, coding and *cis*-regulatory mutations in floral anthocyanin-regulating R2R3-MYBs are believed to contain few negative, pleiotropic effects (Martin et al., 2010), which suggests that they too will have high probabilities of fixation.

Analyses of amino acid substitution rates are consistent with these predictions. In the morning glory genus *Ipomoea* (Convolvulaceae), adaptive amino acid substitutions in the anthocyanin pathway enzymes and transcription factors appear rare. Thus, differences in the ratio of non-synonymous to synonymous substitutions (dN/dS) reflect variation in constraint among proteins. Among species of *Ipomoea*, it has been shown that floral anthocyanin-regulating R2R3-MYB transcription factors have significantly greater dN/dS ratios than the pathway enzymes or the bHLH and WDR transcription factors that control floral anthocyanin pigmentation (Lu and Rausher, 2003; Chang et al., 2005; Streisfeld and Rausher, 2007; Toleno et al., 2010; Streisfeld et al., 2011). Therefore, to the extent that patterns in *Ipomoea* are generalizable and that variation in constraint is associated with differences in pleiotropy, these results are consistent with our predictions that coding mutations in floral-specific R2R3-MYBs should have few deleterious pleiotropic effects.

To provide a crude approximation of the mutation spectrum underlying losses in floral pigmentation, Streisfeld and Rausher (2011) surveyed the literature for examples of spontaneous flower color mutations that were preserved by the horticultural industry and subsequently genetically characterized. Mutations in all of the pathway genes and transcription factors occurred readily, with the only bias being that *cis*-regulatory mutations in pathway enzymes occurred less frequently than others. This is presumably due to differences in mutation target size, as the functional units of *cis*-regulatory regions may be much smaller than the genes they regulate. Within-population polymorphisms likely have arisen relatively recently, which indicates that they have not passed through many selective filters. Therefore, repeated examples involving segregating flower color variants are likely to recapitulate this mutation spectrum.

By contrast, fixed differences in flower color between populations or species should preferentially include *cis*-regulatory mutations in any of the genes capable of reducing pigment intensity, including members of the Anthocyanin Biosynthetic Pathway (ABP) or associated transcription factors. However, coding mutations should only exhibit a fixation bias in cases where the mutation has tissue specific effects, such as what is presumed for anthocyanin-regulating R2R3-MYBs.

### Empirical examples

Based on our predictions, we now turn to the observed empirical data on genetic characterizations of losses of floral pigment intensity within and between populations (Table 1). While the sample sizes in each category are small, the limited data that do exist are all consistent with our predictions based on differences in deleterious pleiotropy.

**Table 1:** **Species for which the genetic basis of altered floral pigment intensity have been characterized**.

**Taxon**	**Gain (G) or Loss (L)**	**Segregating (S) or fixed (F)**	**Derived phenotype**	**Locus**	**Gene**	**Mutation**	**References**
*Petunia axillaris*	L	F	White^a^	*AN2*	R2R3-MYB (tf)	Coding^d^	Quattrocchio et al., 1999
*Antirrhinum majus* ssp. *striatum*	L^c^	F	Yellow^a^	*ROSEA*	R2R3-MYB (tf)	?	Whibley et al., 2006
*Antirrhinum latifolium*	L^c^	F	Pale^a^	*ROSEA*	R2R3-MYB (tf)	?	Schwinn et al., 2006
*Antirrhinum meonanthemum*	L^c^	F	Pale^a^	*ROSEA*	R2R3-MYB (tf)	?	Schwinn et al., 2006
*Antirrhinum molle*	L^c^	F	Pale^a^	*ROSEA*	R2R3-MYB (tf)	?	Schwinn et al., 2006
*Antirrhinum graniticum*	L^c^	F	Pale^a^	*ROSEA*	R2R3-MYB (tf)	?	Schwinn et al., 2006
*Antirrhinum mollissimum*	L^c^	F	Pale^a^	*ROSEA*	R2R3-MYB (tf)	?	Schwinn et al., 2006
*Ipomoea purpurea*	L	S	White^a^	*W*	R2R3-MYB (tf)	Coding^d^	Chang et al., 2005
*Ipomoea purpurea*	L	S	White^a^	*A*	CHS^e^	Coding^d^	Habu et al., 1998
*Parrya nudicaulis*	L	S	White^b^	*?*	CHS^e^	cis^f^	Dick et al., 2011
*Mimulus lewisii*	L	S	White^a^	*MlDfr*	DFR^e^	Coding^f^	Wu et al., in review
*Mimulus aurantiacus*	G	F	Red^a^	*MaMyb2*	R2R3-MYB (tf)	cis^d^	Streisfeld et al., 2013
*Mimulus cupreus*	G	F	Orange^a^	*PLA1*	R2R3-MYB (tf)	?	Cooley et al., 2011
*Mimulus variegatus*	G	F	Purple^a^	*PLA2*	R2R3-MYB (tf)	?	Cooley et al., 2011
*Mimulus cardinalis*	G	F	Dark red^b^	*ROI1*	R3-MYB (tf)	cis^d^	Yuan et al., 2013
*Phlox drummondii*	G	F	Dark red^b^	*MYB*	R2R3-MYB (tf)	cis^d^	Hopkins and Rausher, 2011

a Qualitative change in pigment intensity.

b Quantitative change in pigment intensity.

c Phylogenetic analyses equivocal with respect to directionality and number of independent transitions.

d Validated via transgenic and/or allele specific expression.

e Gene encodes an ABP enzyme.

f Inferred from sequence and/or expression analyses.

? Data either not reported or unknown.

(tf) Gene encodes a transcription factor.

#### Fixed differences

As noted above, differences in the magnitude of deleterious pleiotropy among mutation categories should result in the preferential fixation of *cis*-regulatory mutations in all genes and coding mutations in R2R3-MYB transcription factors. Even though losses of floral pigment appear to be more frequent that gains (Rausher, 2008), surprisingly, there is only one unambiguous example in the literature of a fixed loss of floral pigmentation where the genetic basis of the change has been characterized. Additional examples have been shown either to consist of gains of floral pigmentation (see below) or the ancestral state is not resolved because of limited phylogenetic signal (e.g., *Antirrhinum*).

The one example where data on the direction are clear involves sister species of *Petunia* in their native South America. *P. integrifolia* has purple flowers, while its sister species *P. axillaris* has evolved white flowers via a loss of anthocyanin pigmentation (Quattrocchio et al., 1999). This change in flower color is due to one of five coding mutations within the *AN2* gene, which encodes an R2R3-MYB transcription factor (Quattrocchio et al., 1999), and is associated with a transition to hawkmoth pollination that occurred late in the speciation process (Hoballah et al., 2007).

Among the European clade of snapdragons (*Antirrhinum*), flower colors range from multiple, fully pigmented species to several species with pale to white or yellow flowers with greatly reduced anthocyanin content. Unfortunately, the phylogenetic relationships in this group are poorly resolved (Vargas et al., 2009), which makes it difficult to determine how many times and in which direction this trait evolved. Even though it has been argued that intense floral pigmentation reflects a gain from pale-flowered ancestors (Shang et al., 2011), other phylogenetic analyses remain equivocal (Wilson and Hudson, 2011). We therefore present these data as losses, but acknowledge that further phylogenetic analyses are warranted. Recent developments in next-generation sequencing technologies for resolving difficult evolutionary relationships among closely related groups (Emerson et al., 2010) suggest that these approaches would be ideal for establishing the directionality of flower color transitions among snapdragon species.

Based on the results of genetic crosses with laboratory accessions of *A. majus*, Schwinn et al. (2006) determined that an allele at the *Rosea* locus encoding two R2R3-MYB proteins is responsible for white or greatly reduced floral anthocyanin pigmentation in *A. granticum, A. molle*, and *A. mollissimim* (all contain the *ros* allele). In addition, a mutation in a second allele at this same locus (*ROS*^*El*^) is responsible for the evolution of reduced floral pigmentation in two additional species (*A. latifolium* and *A. meonanthemum*) (Schwinn et al., 2006). Moreover, the yellow-flowered *A. majus striatum* also contains the *ros* allele, while the magenta-flowered *A. majus pseudomajus* contains a functioning copy of *ROS* that is capable of activating the anthocyanin pathway enzymes (Whibley et al., 2006). Thus, depending on the true phylogenetic relationships among these species, at least two, and as many as six occurrences of reduced anthocyanin pigmentation are associated with mutations in R2R3-MYBs. Alternatively, if fully-pigmented flowers represent the derived state in this group (as suggested by Shang et al., 2011), then this suggests the possibility of multiple independent gains of floral pigment intensity. However, the genetic basis for these fully-pigmented transitions have not been established to determine whether these examples represent convergence at the genetic level involving multiple gains of pigmentation.

Moreover, in the recently radiated columbines, six independent losses of floral pigmentation have been described (Whittall et al., 2006). Genetic crosses indicate that white flowers in the *Aquilegia chrysantha* clade segregate as a single Mendelian locus (Prazmo, 1961). In addition, gene expression analyses reveal downregulation of multiple pathway enzymes, suggesting that altered function or expression of a transcription factor is likely responsible. However, because the precise genetic change has not been identified, it is unknown whether a mutation in an R2R3-MYB protein or some other regulator of the pathway enzymes is involved. Additional examples of fixed losses in floral pigment intensity in other white-flowered *Aquilegia* as well as *Ipomoea* species also suggest that reductions in transcriptional regulation of the pathway enzymes are the likely cause for white flowers (Durbin et al., 2003; Whittall et al., 2006). However, the researchers have not ruled out the possibility that these regulatory changes occurred secondarily to other mutations that were causally related to the change in flower color. Taken together, the data for fixed losses of floral pigment intensity do support the prediction that coding mutations in any of the genes except those encoding R2R3-MYBs are unlikely to fix between species because of their deleterious pleiotropic effects. We suspect that as more data are collected, examples of *cis*-regulatory mutations in pathway genes will also be responsible for fixed losses in floral pigmentation in proportion to their mutation rate.

#### Within-population polymorphism

There are now four examples of the genetic characterization of loss of pigment intensity mutations segregating within populations (Table 1). Two of these consist of exceptionally rare white-flowered variants in populations of otherwise intensely pigmented individuals. In the normally purple-flowered *Ipomoea purpurea*, white flowers at the *A*-locus are caused by an insertion in the coding region of the *Chs* gene, which encodes the first enzyme of the ABP (Habu et al., 1998). The white-flowered, *a*-allele is never found at a frequency above 0.005 (Coberly and Rausher, 2008). The rarity of this allele in populations has been attributed to experimentally validated negative pleiotropic effects that influence floral production, heat tolerance, and fecundity even though a significant transmission advantage through self-pollen should allow the allele to increase in frequency (Coberly and Rausher, 2003; Fehr and Rausher, 2004; Coberly and Rausher, 2008).

In addition, data from *Mimulus lewisii* suggest that a frameshift in the second exon of *MlDfr* (encoding the pathway enzyme DFR) is responsible for the evolution of rare white flowers in a population in Oregon (Wu et al. in review). The *white* allele at *MlDfr* also appears to be quite rare, with an estimated allele frequency of 0.026. No information currently exists about possible pleiotropic consequences of the *MlDfr* mutation in *M. lewisii*. However, based on *Dfr*'s position in the anthocyanin pathway, a null mutation would prevent the synthesis of not only anthocyanin pigments, but also several flavonoid compounds that are produced downstream of DFR, including the proanthocyanidins (or condensed tannins). These compounds have been shown to be important in protecting plants from both microbial pathogens and insect herbivores (Dixon et al., 2005). Thus, because these rare variants likely have not passed through substantial selective filters, the fact that mutations appear in nature that harbor deleterious pleiotropic effects suggests that these mutations will remain rare or eventually will be lost from the population. These results are therefore consistent with our prediction that a broader spectrum of mutations will be found among segregating anthocyanin losses. We expect that as more examples are characterized, the distribution of mutations among categories will reflect the underlying mutation spectrum.

The other two examples of segregating polymorphism within populations consist of variants that are found more commonly but have not fixed in populations. In *Ipomoea purpurea*, a second white-flowered mutant also segregates within populations in the southeastern USA and is encoded at the *W*-locus due to a deletion in the coding region of an R2R3-MYB transcription factor (*IpMyb1*) (Chang et al., 2005). In contrast to the exceptionally rare *a*-allele that also confers white flowers, the white allele (*w*) at the *W*-locus can be found at frequencies up to 0.5 in nature. Furthermore, despite extensive investigation, no deleterious pleiotropic effects on fitness have been associated with the *w*-allele (Rausher and Fry, 1993), which further supports the prediction that coding mutations in R2R3-MYBs have few deleterious pleiotropic effects.

Finally, in an arctic mustard (*Parrya nudicaulis*) with a purple-white polymorphism that varies clinally across Alaska, white flowers appear to be due to a *cis*-regulatory mutation in *Chs* (Dick et al., 2011). Although it is unknown whether differential fitness associated with the purple or white alleles occurs across this cline, it is intriguing that white flowers can occur at frequencies up to 24% and are caused by a *cis*-regulatory mutation. However, because *Chs* is the first enzyme necessary in the anthocyanin pathway, reduced expression of this gene in the floral tissue would also prevent the production of any of the downstream flavonoids (e.g., aurones, flavonones, flavonols, etc.; see Figure 1). Therefore if any of these products play significant physiological roles directly in the flower, there may be negative pleiotropic consequences of the mutation conferring white flowers.

While the sample size is small, the pattern emerging from these examples suggests that different types of mutations are targeted at alternate scales. The one confirmed example of a fixed loss involves a coding mutation in an R2R3-MYB. In addition, the partial genetic evidence in the remaining examples of fixed losses (e.g., *Aquilegia*) suggests that reduced pigmentation is caused by decreased expression levels as opposed to losses of enzymatic function. While the precise nature of these genetic changes is unclear, it is worth noting they do not implicate coding mutations in pathway enzymes. Moreover, the mutations responsible for the two intermediate frequency polymorphisms are consistent with the notion that these alleles have risen to higher frequency because they have few deleterious pleiotropic effects. By contrast, the two examples of rare polymorphisms are caused by coding mutations that eliminate enzyme function. This supports the notion that of the broad suite of mutations that arise and segregate in populations, only a small set of them will become fixed between populations.

## Evolutionary gains of floral pigment intensity

Gains of novel traits are expected to occur less frequently than losses because there are typically more ways to break a pathway than to restore it. This argument essentially states that the mutation spectrum for losses will be greater than the mutation spectrum for gains. While this may often be true (see below), some traits may be easier to gain than others. For example, with the notable exception of the Caryophyllales, nearly all higher plants produce anthocyanin in vegetative tissue (Brockington et al., 2011), where it is involved in a variety of physiological functions (Winkel-Shirley, 2002; Grotewold, 2006; Rausher, 2008). As a result, the ABP and its regulation are conserved through large spans of evolutionary time, regardless of whether pigment is produced in flowers. Consequently, gaining floral anthocyanins in a lineage usually does not require the restoration of a non-functioning pathway, but instead a simple shift in regulation of the ABP genes into this expression domain. Therefore, gains of floral anthocyanin pigmentation may occur more readily than gains in other phenotypic traits.

Although a mutation in any of the four categories listed above is capable of generating a loss of floral anthocyanins, the deployment of ABP gene regulation into the flower will likely be restricted to mutations in transcription factors (Martin et al., 2010). In lineages where anthocyanins were previously absent, the novel recruitment of the ABP in flowers can be accomplished via activating mutations in members of the MBW complex. Because of their functional specificity and limited deleterious pleiotropic effects, *cis*-regulatory mutations in R2R3-MYBs are the most likely mutations to produce these co-option events. Alternatively, increased pigment intensity can occur in lineages where anthocyanins are already present. This can occur either by removing the repression of a transcription factor that inhibits proper expression of the ABP genes in floral tissue or by quantitatively increasing expression of ABP genes in flowers.

As with losses, the deleterious pleiotropic effects of the various mutations capable of increasing floral pigmentation are expected to be the major factor determining which mutations are targeted by natural selection. However, because the range of possible mutations that can contribute to a gain is less than a loss, it is unlikely that natural selection will have the opportunity to simultaneously sample multiple mutations and target the one with the fewest pleiotropic effects. Instead, the predictions for gains are more contingent on the relative balance between the strength of selection favoring the novel flower color and the negative pleiotropic effects of that mutation. Therefore, in the case of anthocyanin gains, it may be common for a mutation with deleterious effects to fix in a population if the strength of natural selection favoring a novel flower color outweighs the negative pleiotropic effects of that mutation.

Flower color evolution traditionally has been viewed as driven primarily by variation in pollinators (Fenster et al., 2004). However, it is now appreciated that non-pollinator agents of selection can also play significant roles (Armbruster, 2002; Strauss and Whittall, 2006). For example, as a consequence of the multifunctional ABP, flavonoids that play important physiological roles for the plant have the potential to result in indirect changes in floral anthocyanin pigment intensity. Therefore, even though multiple agents of selection may be responsible for differences in flower color, in attempting to make the connection between flower color transitions and the spectrum of negative pleiotropy that can arise, we find it illustrative to first consider cases in which gains in anthocyanin are being directly favored (by whatever agent). Thus, for simplicity, we present the following scenarios as if pollinators alone impose direct selection on flower color transitions, with negative pleiotropic consequences arising as a result of altered vegetative physiological traits under the control of the same genes. We first describe the various mechanisms that can lead to evolutionary gains in floral pigmentation and then determine how the strength of selection on this phenotype affects the types of mutations that result in gains in flower color in nature.

### Co-option of the anthocyanin pathway

The co-option of transcription factors and subsequent redeployment of conserved developmental pathways is predicted to be a prime mechanism of evolutionary innovation and adaptation (True and Carroll, 2002; Wagner, 2011). Moreover, gene duplication is thought to play a vital role in the functional diversification of eukaryotes (Ohno, 1970; Innan and Kondrashov, 2010), which may provide the raw materials necessary for co-option to occur. The relationships between the processes of duplication and co-option have been examined extensively in the evolution of floral symmetry and the underlying MADS-box transcription factors (Kramer et al., 1998; Rosin and Kramer, 2009).

Although many of these possibilities are yet to be explored in floral anthocyanin evolution, the R2R3-MYBs have displayed an especially rich history of gene duplication both early in the evolution of land plants and in more contemporary radiations (Dias et al., 2003; Feller et al., 2011), making them interesting candidates for potential co-option events. Recently duplicated R2R3-MYBs frequently display signatures of positive selection (Jia et al., 2003), and functional divergence may often follow duplication events (e.g., Haberer et al., 2004; Baumann et al., 2007). Several non-mutually exclusive models exist to explain how a recently duplicated gene may become differentiated and maintained in the genome (Lynch and Conery, 2000; Innan and Kondrashov, 2010), and the precise history of duplication and post-duplication divergence may impact the degree of pleiotropy resulting from mutations that give rise to increased floral anthocyanins.

**Figure 2:**
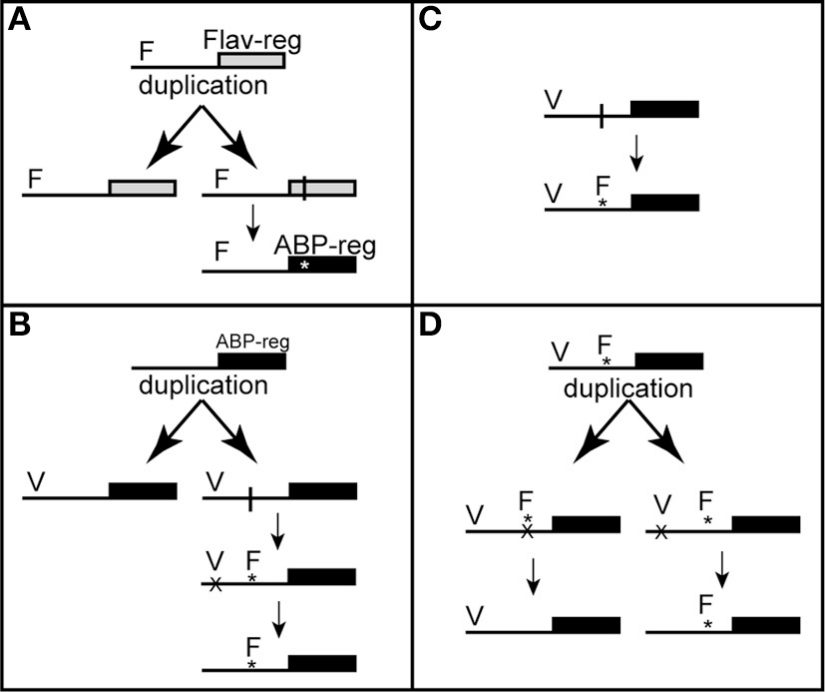
**Pathways of co-option leading to gains in floral anthocyanins.** Solid boxes represent coding regions of R2R3-MYB transcription factors, and horizontal lines represent their *cis*-regulatory elements. Within these regions, “F” denotes a floral *cis*-regulatory element, while “V” indicates a vegetative element. Vertical lines denote mutation locations and asterisks the resulting substitution. An “X” indicates a loss of function mutation. **(A)** Gene duplication followed by co-option via neofunctionalization. An R2R3-MYB that regulates some other product in flowers (such as flavonoids) duplicates. A coding mutation in the DNA binding domain of one copy results in a transition to ABP regulation. **(B)** Gene duplication followed by co-option and specialization. Duplication of an R2R3-MYB that regulates vegetative anthocyanins acquires a *cis*-regulatory mutation in one copy that introduces a floral specific element. Specialization occurs if the copy with the floral *cis*-element loses its vegetative expression. **(C)** Co-option occurs without gene duplication when a mutation introduces a floral *cis*-element into a pre-existing R2R3-MYB that regulates vegetative anthocyanins. **(D)** Co-option of a pre-existing R2R3-MYB followed by gene duplication. After the co-option of the ABP for floral anthocyanins, vegetative and floral anthocyanins are favored differently by selection. In these cases, gene duplication may be advantageous to reduce this constraint. Post duplication deletions of the alternate *cis*-elements then produce specialization.

While neofunctionalization of a recently duplicated gene is expected to be relatively rare [but see (Keeling et al., 2008)], duplication of an R2R3-MYB that controls a related function, such as non-anthocyanic floral flavonoids, may be followed by coding mutations in one gene copy that alters the DNA binding domain of the transcription factor (Figure 2A). Conceivably, this could result in novel ABP regulation in the flower. However, because all known anthocyanin regulating R2R3-MYBs form a monophyletic group (Stracke et al., 2001; Dubos et al., 2010), it appears that duplication of non-anthocyanin R2R3-MYBs is an unlikely mechanism for floral pigment gains.

A more likely scenario might be the duplication of an R2R3-MYB that already regulates the ABP in another tissue, followed by a *cis*-regulatory mutation in one copy that increases expression in the flower (Figure 2B). The nature of this altered expression determines the potential pleiotropic impacts of the mutation. For example, the duplication process may eliminate necessary *cis*-regulatory machinery, such as an element required for expression in vegetative tissue. Subsequent *cis*-regulatory mutations in this copy may then allow for its specialized expression in flowers. However, an R2R3-MYB may also be duplicated with its complete suite of *cis*-elements, followed by a gain of an element that allows for floral expression. Unless subsequent specialization occurs to eliminate vegetative expression in this copy, this mutation could be deleterious if it negatively alters the vegetative phenotype. Even though perfect specialization of post-duplication gene copies is thought to evolve only rarely (Simon et al., 2007; Guillaume and Otto, 2012), the functional specialization of a redundant gene copy should have minimal impacts on fitness. Therefore, any mutation that confers a high degree of floral specialization in one of the gene copies should have few pleiotropic effects.

Anthocyanin may also be gained in floral tissue in the absence of gene duplication by the co-option of a pre-existing R2R3-MYB that regulates the ABP elsewhere in the plant (Figure 2C). As an example, an existing R2R3-MYB that induces leaf anthocyanin expression during stress (Chalker-Scott, 1999; Winkel-Shirley, 2002) may experience a *cis*-regulatory mutation that increases its expression levels in floral tissue, leading to positive regulation of the ABP in the flower. This could occur via the introduction of a new *cis*-regulatory element by recombination or through alterations to the existing elements that expand the expression domain of that protein (Flagel and Wendel, 2009). In this latter situation, the R2R3-MYB must simultaneously increase the amount of anthocyanin in the flower while also performing its original vegetative function. Therefore, the pleiotropic effects of mutations that result in the novel recruitment of floral anthocyanin will depend on how much the original vegetative phenotype is altered. Thus, mutations that result in floral expression without altering vegetative function will be most favored by natural selection.

The process of gene duplication may also follow co-option rather than precede it. For example, if an R2R3-MYB has vegetative physiological functions, and it is co-opted to serve as a floral anthocyanin regulator, genetic correlations between these functionally divergent traits may lead to an adaptive conflict due to opposing selective pressures (Figure 2D). In these cases, gene duplication may be favored to release populations from the constraints imposed by employing a single copy of the transcription factor [e.g., Escape from Adaptive Conflict; (Des Marais and Rausher, 2008)]. However, in the evolutionary history of R2R3-MYBs, the most extensive duplication occurred early in the history of land plants, leading to the high copy number observed in extant taxa (Feller et al., 2011). Under the Duplication-Degeneration-Complementation (DDC) model (Force et al., 1999), it is expected that many gene copies retain some level of functional redundancy that may persist in the genome for extended periods. Co-option of the ABP into floral tissue may make use of this ample supply of partially redundant R2R3-MYBs in plant genomes, as their abundance may make this the evolutionary path of least resistance. Regardless, this possibility illustrates how the process of gene duplication and subsequent co-option may be intimately related, even when the two processes are temporally separated by long time spans.

### Non-co-optive gains in floral anthocyanin

While the co-option of R2R3-MYBs suggests that anthocyanins are recruited to flowers due to the evolution of a novel expression domain, in some cases gains of anthocyanins may occur without co-option. For example, anthocyanins may be lacking in flowers due to direct inhibition of ABP expression by a repressor. In these cases, anthocyanin gains could be caused either by a *cis*-regulatory mutation that decreases the expression of the repressor or by a coding mutation in the repressor that results in reduction or elimination of the protein's affinity for its target loci. Even though micro RNAs and other short interfering RNA have the potential to repress floral anthocyanins in various tissues (Rajagopalan et al., 2006), it is interesting to note that both R2R3-MYB and R3-MYB proteins have been shown to repress anthocyanins and other flavonoids in vegetative tissues (Quattrocchio et al., 2006; Matsui et al., 2008; Dubos et al., 2010; Albert et al., 2011). As with co-option, the specificity of the mutation involved determines the potential for pleiotropy. If a protein that actively represses anthocyanins is expressed only in the flower, coding and *cis*-regulatory mutations would have minimal pleiotropic effects. However, if the repressor is also involved in physiological processes, such as reducing anthocyanin expression in order to facilitate production of other flavonoids in vegetative tissue, then a range of effects are possible. However, because the full spectrum of anthocyanin pathway repression is not yet established, predictions are difficult to formulate.

Co-option is also not necessary when anthocyanins are already present in the flower, and the gain in pigmentation represents a quantitative increase in intensity rather than a qualitative transition between absence and presence. In these cases, increases in floral anthocyanin content also may be achieved by *cis*-regulatory mutations in whichever ABP genes or transcription factors are limiting flux down the pathway. If multiple mutations are possible, the pleiotropic consequences of these mutations will dictate which ones are targeted by selection.

### Experimental predictions

Among the various mechanisms that can result in increased floral anthocyanins, many of the mutations can result in increased pigmentation of vegetative tissue as a by-product of floral expression. Because one of the vegetative roles of anthocyanin is to shield plant cells from too much light during periods of stress (Chalker-Scott, 1999; Winkel-Shirley, 2002; Grotewold, 2006), a plant carrying this type of mutation may experience reduced photosynthetic capacity. This presents an obvious cost, and the mutation may never rise to fixation or will only occur in specific habitats. Therefore, an anthocyanic morph segregating within a population is expected to fix if favored by pollinators unless the mutation exhibits such deleterious pleiotropic effects that the direct fitness benefits from pollinators are offset by its secondary impacts. Regardless, the presence of a segregating polymorphism suggests that these variants have yet to pass through substantial selective filters, implying that the underlying mutation spectrum for increased floral anthocyanin content would be a good predictor of the types of mutations that occur in nature.

In cases where fixed gains in floral anthocyanins involve co-option of the ABP, natural selection is expected to favor *cis*-regulatory mutations in the R2R3-MYB copy that has the lowest total impact on plant fitness. The mechanism by which this occurs will depend on the frequency of gene duplication and the mutation spectrum for gains in floral pigmentation. Because R2R3-MYBs already occur in high copy number in plant genomes (Feller et al., 2011), several copies are available for potential co-option events. Therefore, a possible mutation bias associated with *cis*-regulatory mutations in pre-existing R2R3-MYBs, coupled with a predicted fixation bias due to their few deleterious pleiotropic effects, suggests this is the most likely route for these transitions. However, recent gene duplication followed by specialization should not be overlooked, particularly for taxa that have recently undergone whole genome duplication via polyploidization.

For fixed gains where co-option is not responsible, qualitative shifts from an unpigmented ancestor are unlikely to consist of activation mutations, but should involve loss-of-function mutations that eliminate repression. This results in a limited mutation spectrum for this type of transition. Similarly, quantitative increases in pigment intensity often will not involve co-option, but in these cases the mutation spectrum appears broader than for qualitative shifts. Here, quantitative increases in pigmentation could be due to losses of a repressor or activation mutations at rate-limiting steps. The relative frequency of these alternatives will depend to a large extent on the combined strength of selection by pollinators for the more intense flower color and the negative effects of pleiotropy.

### Empirical examples

While evolutionary gains of anthocyanins are assumed to be less frequent than losses (Perret et al., 2003; Rausher, 2008), five putative examples exist in the literature that have received genetic and molecular scrutiny (Table 1). Interestingly, four come from the genus *Mimulus*. Although flower color transitions are relatively common in this group, this taxonomic bias more likely arises from the long history of ecological and evolutionary genetic work in the genus (Wu et al., 2008), rather than some intrinsic feature of the genus that makes evolutionary gains more common than in other groups. We also discuss the increase in floral anthocyanin intensity in the classic model for reproductive character displacement via reinforcement, *Phlox drummondii* (Levin, 1985). These examples provide a limited glimpse into the predictability of this phenotypic transition; indeed, no examples of segregating polymorphisms involving gains have been characterized genetically. Additional examples of putative gains in floral anthocyanin are known in nature (e.g., Armbruster, 2002; Kay et al., 2005), providing ample opportunities to experimentally pursue these hypotheses.

#### Mimulus aurantiacus subsp. puniceus and M. aurantiacus subsp. australis

**Figure 3:**
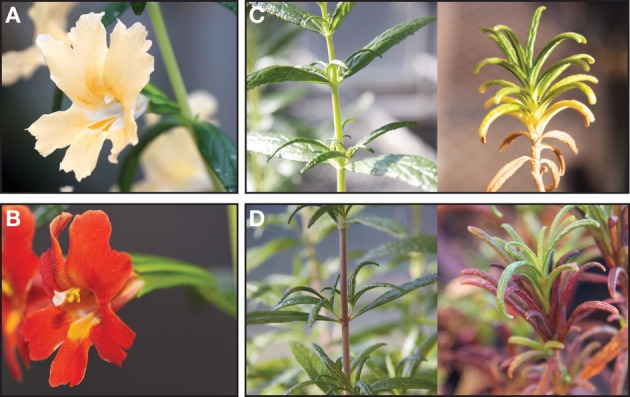
**An example of a gain in floral anthocyanin via co-option of an R2R3-MYB in *Mimulus aurantiacus*. (A)** The yellow flowers of *M. australis* contain no detectable anthocyanin in floral tissue. **(B)** A *cis*-regulatory mutation in *MaMyb2* causes the derived red-flowered phenotype observed in *M. puniceus*, and strong divergent selection on flower color by pollinators has driven the fixation of the red and yellow alleles in nature (Streisfeld et al., 2013). **(C)** Although anthocyanins are present in vegetative tissue in *M. australis*, they are typically found at low concentrations. **(D)** In contrast, vegetative anthocyanins are often induced to high levels in *M. puniceus*, consistent with the possibility that negative pleiotropic effects of the co-option event have been overcome by strong selection by pollinators. The genetics of the relationship between floral and vegetative anthocyanins is currently under investigation.

The section Diplacus in the genus *Mimulus* is a radiation of woody perennial shrubs found in chaparral communities throughout the California floristic province (Grant, 1924). Most members of the section have yellow or orange flowers that completely lack anthocyanin; however, red flowers that contain anthoycanins are derived in *Mimulus aurantiacus* subsp. *puniceus* (hereafter *M. puniceus*) (Figure 3). In the region where *M. puniceus* and the yellow-flowered, *Mimulus aurantiacus* subsp. *australis* (hereafter *M. australis*) meet, the two taxa form a hybrid zone (Beeks, 1962; Grant, 1981). Despite the opportunity for gene flow across the hybrid zone, flower color differences are fixed outside the region of contact (Streisfeld and Kohn, 2005). The red-flowered *M. puniceus* is pollinated almost exclusively by hummingbirds, while the yellow-flowered *M. australis* is primarily pollinated by hawkmoths, demonstrating that divergent selection across the hybrid zone maintains differences in flower color (Streisfeld and Kohn, 2005, 2007).

Genetic analysis revealed two loci responsible for this gain in flower color (Streisfeld and Rausher, 2009). Gene expression and gene knockdown approaches have demonstrated that a *cis*-regulatory mutation in an R2R3-MYB gene (*MaMyb2*) positively regulates at least three ABP enzymes in *M. puniceus* flowers, resulting in red flowers (Streisfeld et al., 2013). Flower color differences between the subspecies also map to a second R2R3-MYB (*MaMyb1*) that is unlinked to *MaMyb2*, but its function has yet to be characterized (Streisfeld and Rausher, 2009).

Based on the nature of the major genetic change underlying the fixation of red flowers in *M. puniceus* (an activating *cis*-mutation in *MaMyb2*), we can infer that co-option of the ABP into flowers is responsible for this transition. Moreover, because *M. puniceus* and *M. australis* both harbor *MaMyb2* loci in their genomes, this suggests that the co-option of the ABP in flowers did not involve a recent duplication of *MaMyb2* in the lineage leading to *M. puniceus*. Therefore, among the above mechanisms that can generate co-option, we can rule out neofunctionalization and duplication followed by specialization (Figures 2A,B). Instead, this appears to be a case in which the diversity of R2R3-MYBs already present in the genome allowed for the co-option of the ABP by *cis*-regulatory mutations in an existing gene (as in Figure 2C).

Moreover, *M. puniceus* and *M. australis* commonly differ in their induction of vegetative anthocyanins due to stress (Figures 3C,D), which suggests that the *cis*-acting mutation in *MaMyb2* that gave rise to red flowers also may influence vegetative pigmentation. While likely minor, if pleiotropic effects of this mutation in *MaMyb2* exist, they may have been overcome by selection by pollinators. Thus, co-option of the ABP to flowers appears to be associated with an adaptive shift due to altered pollinator preferences. Additional questions remain in this system, which may help reveal how this co-option event occurred. For example, do genetic correlations between vegetative and floral anthocyanins impact the efficacy of selection? What is the evolutionary history of the causal mutation that led to co-option? Finally, does the causal mutation in *MaMyb2* have the fewest pleiotropic effects among the other R2R3-MYBs found in the genome, or was it simply the mutation that occurred under a specific selective scenario?

#### The chilean mimulus

**Figure 4:**
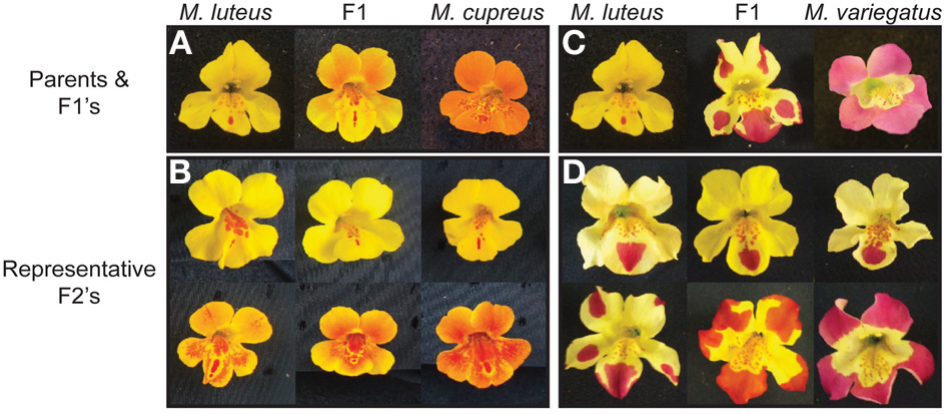
**Two trajectories for floral anthocyanin gains in the Chilean *Mimulus*. (A)** *M. luteus* has yellow flowers with red anthocyanic spots in the throat, typical of other closely related species in the genus. *M. cupreus* has gained anthocyanin throughout the corolla, giving it a red-orange color. **(B)** F2's from a cross between *M. luteus* and *M. cupreus* show that petal lobe anthocyanins segregate independently from variation in anthocyanic throat markings (Cooley and Willis, 2009). **(C)** *M. variegatus* is a second taxon that independently gained floral anthocyanins (Cooley et al., 2011). **(D)** Segregating variation in F2's between *M. luteus* and *M. variegatus* suggests that variation in anthocyanins may occur via repatterning of the spot on the lower-middle petal. Extensive variation in size and shape of this marking is evident, including examples where expansion of spots cover the entire distal half of each petal. All photos provided by Arielle Cooley.

*Mimulus* section Simiolus, which includes the widely studied *Mimulus guttatus*, is a group of primarily yellow-flowered plants that often exhibits relatively small anthocyanic spots on the lower part of the corolla (Beardsley et al., 2004; Wu et al., 2008). Nested within this section is a small radiation of *Mimulus* species native to central Chile. All of the members of this group are presumed to be recent tetraploids with a base chromosome number of *n* = 32, relative to the *n* = 14 typically found in the closely related *M. guttatus* and *M. glabratus* species complexes (Vickery, 1995). Some of the Chilean *Mimulus* exhibit the yellow-flower color phenotype typical of section Simiolus (e.g., *M. luteus* subsp. *luteus;* hereafter *M. luteus*), while additional members of the radiation have gained anthocyanin throughout the petal lobes (e.g., *M. cupreus;* Figure 4A, *M. luteus* subsp. *variegatus;* hereafter *M. variegatus*; Figure 4C). While variation in the size and shape of the anthocyanic spots in *M. luteus* has been shown to affect selection by pollinators to some extent (Medel et al., 2003), pollinator preference does not seem to be an important factor in driving the evolution of highly anthocyanic flowers in *M. cupreus* or *M. variegatus* (Cooley et al., 2008). Nevertheless, these phenotypic transitions raise the possibility that the history of polyploidy in the group may have facilitated gains in anthocyanin via specialization of gene duplicates. Alternatively, it is possible that the petal spots present in the ancestor were simply re-patterned to the entire corolla in the pigmented species, which would represent an alternate mechanism for altering floral color from pre-existing variation.

Genetic crosses between the yellow-flowered *M. luteus* and the anthocyanic taxa (*M. cupreus* and *M. variegatus*) reveal that flower color segregates as a single Mendelian locus in both cases (Cooley and Willis, 2009). However, in a cross between the two anthocyanic taxa, some of the offspring showed no floral pigmentation, indicating the presence of independent loci, and thus independent gains, controlling floral anthocyanins in these species (Cooley and Willis, 2009).

The locus responsible for orange-red flowers in *M. cupreus* co-localizes with a region in *M. guttatus* containing three tightly linked anthocyanin regulating R2R3-MYBs (*MgMyb1-3*). As expected due to recent tetraploidy, Cooley et al. (2011) identified all six of the R2R3-MYBs at this petal lobe anthocyanin locus (*PLA-1*) in *M. cupreus*. Moreover, consistent with one of these R2R3-MYBs controlling the gain of floral anthocyanins, the *M. cupreus* allele at *PLA-1* results in elevated expression levels of ABP genes in segregating lines. In *M. variegatus*, the floral anthocyanin conferring locus (*PLA-2*) is associated with an independent region containing two additional R2R3-MYBs in *M. guttatus* (*MgMyb4-5*). Expression patterns are also consistent with a causal transcription factor encoded at *PLA-2*. Cooley et al. (2011) speculate that *cis*-regulatory mutations in these R2R3-MYBS's cause both anthocyanic phenotypes. However, functional tests are currently lacking, preventing us from determining which, if any, of these R2R3-MYBs are actually responsible for differences in flower color between these species.

In *M. luteus*, the anthocyanin spot on the lower petal lobe can display considerable variation in shape and size in natural populations (Medel et al., 2007). In F2 crosses between this typical yellow-flowered taxon and the highly pigmented *M. variegatus*, the full range of variation in petal size and position exists, with some individuals resembling *M. luteus* with a single large spot on the lower-most petal lobe, while others have spots extending and covering all five petals (Figure 4D) (Cooley and Willis, 2009). These results suggest that rather than co-option of a vegetative R2R3-MYB, the fully-pigmented phenotype in *M. variegatus* may have been caused by repatterning of the size and position of this spot. Unfortunately, the factors that control the formation and positioning of anthocyanin spots or stripes are not well characterized, but have been hypothesized to involve a combination of precise spatio-temporal expression of R2R3-MYBs in the same domain as bHLH transcription factors (Shang et al., 2011; Davies et al., 2012). Therefore, it is possible that a *cis*-regulatory mutation in the *PLA-2* R2R3-MYBs was responsible for the expansion of the spot into additional petals followed by subsequent mutations elsewhere in the genome that extended the size of the spot, leading to the fully pigmented floral tissues in *M. variegatus*. Additional work in this system should focus on characterizing the functional molecular basis for this transition. If the anthocyanic spot seen in *M. luteus* is controlled by an R2R3-MYB with very high tissue specificity, then this transition could have occurred with very few negative pleiotropic effects.

On the contrary, the anthocyanic spots observed in F2's between *M. cupreus* and *M. luteus* are largely invariant (Figure 4B) (Cooley and Willis, 2009). Therefore, *PLA*-1 appears to control variation in intensity throughout the rest of the corolla without affecting the markings that are present in the ancestral form. This suggests that the evolution of floral anthocyanins in *M. cupreus* represents a co-option event, via the recruitment of an R2R3-MYB at *PLA-1* from a vegetative to a floral function. Consistent with this hypothesis, five different vegetative anthocyanin phenotypes in *M. guttatus* have been shown to map to the homologous genomic region that contains *PLA-1* (Lowry et al., 2012). The causal mutation driving increased floral pigmentation in *M. cupreus* could therefore alter a vegetative trait, raising the possibility that negative pleiotropy has been overcome by selection. However, Cooley et al. (2008) show that pollinator preferences do not seem tied to this floral anthocyanin phenotype, which obscures the agent of selection potentially responsible for the fixation of this trait. Moreover, because homologous and homeologous R2R3-MYBs have not been tested functionally in *M. cupreus*, it is unknown whether this co-option event occurred from an R2R3-MYB that preceded the whole genome duplication or arose as a direct consequence of gene duplication through polyploidy followed by specialization. Future work that distinguishes between these hypotheses will provide essential information about the predicted levels of pleiotropy that accompanied this transition.

#### Mimulus lewisii and M. cardinalis

Similar to the *M. aurantiacus* example above, divergent selection exerted by different pollinators has resulted in flower color divergence between *M. lewisii* and *M. cardinalis* (Bradshaw and Schemske, 2003). *M. lewisii* has light pink flowers due to low concentrations of floral anthocyanins and is primarily pollinated by bees. Intensification of floral anthocyanins occurred in the lineage leading to *M. cardinalis*, which is a hummingbird pollinated species that contains dark reddish-orange flowers due to a combination of carotenoids and a quantitative increase in anthocyanin deposition (Bradshaw et al., 1998; Beardsley et al., 2003). The carotenoid locus, *YUP* has been the subject of much previous scrutiny, as the locus is thought to contribute most to variation in pollinator behavior (Hiesey et al., 1971; Schemske and Bradshaw, 1999; Bradshaw and Schemske, 2003). However, in an F2 experimental population, differences in floral anthocyanin content also contributed to pollinator visitation, with a positive relationship between anthocyanin content and hummingbird visitation and a negative relationship with bee visits (Schemske and Bradshaw, 1999). QTL analysis found a single large effect locus that explained roughly 20–30% of the variation in anthocyanin content between the parent species, with the more highly anthocyanic allele recessive to the lighter allele (Bradshaw et al., 1995, 1998).

Because this transition represents a quantitative increase in pigment deposition as opposed to a qualitative one, our predictions suggest that co-option is an unlikely mechanism to explain the elevated anthocyanin content in *M. cardinalis* flowers. Instead, we expect either the loss of a repressor or modifications to the existing regulatory machinery to increase flux down the pathway. Yuan et al. (2013) convincingly demonstrated via transgenic and expression analyses that a *cis*-regulatory mutation results in reduced expression of a gene with sequence similarity to the R3 class of MYB proteins that have been shown to act as repressors in other species. Thus, it appears that loss of a repressor has led to a quantitative increase in floral anthocyanin intensity in *M. cardinalis*, though the precise mechanism for this repression is unknown. Once again the potential for pleiotropic consequences of this mutation rests upon its degree of tissue specificity and the strength of selection favoring the increase in floral anthocyanins. Although this example involves a gain of anthocyanin via the loss of expression of a repressor, the presence of active repression of the ABP suggests that gains of repressor activity may also result in losses of floral anthocyanin. However, while both R3- and R2R3-MYBs have been implicated as repressors of vegetative anthocyanins (Albert et al., 2011; Dubos et al., 2010), we are unaware of examples where gains of repressor function are responsible for evolutionary losses in flower color.

#### Phlox drummondii

As with the above example in *Mimulus lewisii* and *M. cardinalis*, the shift in floral anthocyanins in *Phlox drummondii* involves a quantitative transition from less to more intense anthocyanins. *P. drummondii* and its close relative *P. cuspidata* are involved in a classic case of reproductive character displacement as a result of reinforcement (Levin, 1985). Crosses between *P. drummondii* and *P. cuspidata* reveal strong intrinsic postzygotic isolation, such that selection by pollinators favors a reduced incidence of hybridization when these two species co-occur (Levin, 1985; Hopkins and Rausher, 2012). In regions where *P. drummondii* and *P. cuspidata* are allopatric, they both have pale blue flowers. However, in regions of sympatry, *P. drummondii* has experienced a derived shift in flower color that consists of both an increase in intensity from light to dark flowers and a change in hue from blue to red. We focus here on the shift in anthocyanin intensity, which, in this system, is most important for pollinator visitation (Hopkins and Rausher, 2012).

Because strong selection favors the transition from light to dark pigmentation patterns, we would expect that some negative pleiotropy may be overcome during this floral anthocyanin gain. The genetic and molecular characterization of this transition reveals that a *cis*-regulatory mutation in an R2R3-MYB transcription factor results in dark flowers via positive regulation of several members of the ABP (Hopkins and Rausher, 2011). As in the *M. aurantiacus* system, it does not appear that recent gene duplication was required to drive this gain in intensity, as both allopatric and sympatric populations of *P. drummondii* have alleles at this locus. Co-option is also likely ruled out, as this shift in intensity appears to have been driven by increasing the expression of a pre-existing floral R2R3-MYB rather than enlisting a vegetative locus. Thus, modification to the existing *cis*-regulatory machinery in this R2R3-MYB appears to have resulted in the quantitative increase in floral anthocyanin pigment intensity, which simultaneously resulted in a significant shift in pollinator visitation to complete the speciation process (Hopkins and Rausher, 2012).

## Conclusions

Flower color has been the subject of analysis since the origin of the field of genetics (Mendel, 1866). The ease with which it can be measured, its potential impact on plant fitness, and the characterization of the associated molecular pathways have continued to make this trait an attractive focus of research (Rausher, 2008). Ecological and evolutionary genetic studies of this trait have provided answers to long-standing questions regarding the molecular basis of adaptive traits. Moreover, using a phylogenetic context to examine the genetic basis of repeated phenotypic transitions makes it possible to address central goals of evo-devo research, such as whether genetic evolution is predictable. We have shown that the directionality of evolutionary transitions in flower color, is very likely to impact the precise nature of genetic evolution by altering the mutation spectrum available for gains and losses of floral pigmentation (Table 1). Nevertheless, all but one of the fixed differences in flower color between populations involve *cis*-regulatory or coding mutations in R2R3-MYB proteins. This remarkable pattern of genetic convergence occurs both for losses and gains of floral anthocyanins and can be explained by the amazing diversity and specialization of R2R3-MYB proteins present in the genomes of most flowering plants. Moreover, the fact that a broader set of mutations is evident among segregating polymorphisms further supports the notion that natural selection preferentially and predictably targets those mutations with the fewest deleterious pleiotropic effects.

Although we realize that sample sizes are small and that patterns may change as more examples are characterized, current results are consistent with the predictions that we generated simply based on our knowledge of the ABP and its regulation. However, it is important to note that many of our predictions rest on the assumption that differences in pleiotropy accompany the various mutations capable of altering flower colors. While a reasonable assumption, empirical studies that characterize the extent of pleiotropy of individual mutations and their associated fitness consequences are important next steps for determining the mechanisms that lead to this pattern of genetic convergence (e.g., Von Wettberg et al., 2010).

In conclusion, this review demonstrates how focusing on the repeated evolution of single traits allows us to make mechanistic predictions of the genetic targets of selection responsible for adaptive shifts in phenotype. In addition, because the ABP has conserved physiological roles in vegetative tissue, recurrent gains of flower color are observed in many plant families. Therefore, the evolution of R2R3-MYB transcription factors also can be used to study how conserved developmental networks are co-opted for novel functions, providing invaluable insight into an important source of phenotypic diversity. Finally, even though the long history of studying the anthocyanin pathway and its conserved nature among the angiosperms make transitions in flower color an excellent model for addressing these questions, if repeated transitions in other traits are studied in a similar way, it may be possible to identify general and replicated patterns about the predictability of genetic evolution. Therefore, this study underscores the need to continue dissecting the genetic basis of convergent evolution across phylogenetic scales, including the further development of flower color transitions as an essential model trait in evo-devo.

### Conflict of interest statement

The authors declare that the research was conducted in the absence of any commercial or financial relationships that could be construed as a potential conflict of interest.
